# Comprehensive bioinformatics and *in vivo* validation reveal key molecular drivers of diabetic nephropathy progression

**DOI:** 10.3389/fendo.2025.1654401

**Published:** 2025-09-03

**Authors:** Yakun Yang, Xingyu He, Wei Liu, Lin Mu, Shuo Wang

**Affiliations:** ^1^ Department of Pharmacology, The Key Laboratory of Neural and Vascular Biology, Ministry of Education, The Key Laboratory of New Drug Pharmacology and Toxicology, Collaborative Innovation Center of Hebei Province for Mechanism, Diagnosis and Treatment of Neuropsychiatric Diseases, Hebei Medical University, Shijiazhuang, Hebei, China; ^2^ Key Laboratory of Kidney Diseases of Hebei Province, Department of Pathology, Center of Metabolic Diseases and Cancer Research, Institute of Medical and Health Science, Hebei Medical University, Shijiazhuang, Hebei, China; ^3^ Department of Nephrology, The Second Hospital of Hebei Medical University, Shijiazhuang, Hebei, China

**Keywords:** diabetic nephropathy, bioinformatics analysis, key genes, inflammation, machine learning

## Abstract

**Background:**

Diabetic nephropathy is a leading cause of end-stage renal disease worldwide, characterized by progressive glomerulosclerosis, chronic inflammation, and extracellular matrix (ECM) accumulation. Despite advances in clinical management, the underlying molecular mechanisms remain incompletely understood, and reliable biomarkers for early diagnosis and targeted therapy are still lacking.

**Methods:**

To identify candidate molecular genes associated with DN, we conducted an integrative bioinformatics analysis combining transcriptomic profiling, weighted gene co-expression network analysis, protein–protein interaction network construction, and machine learning-based feature selection. The biological relevance of candidate genes was validated using human renal biopsy specimens and two diabetic mouse models. Gene set enrichment analysis was performed to uncover associated functional pathways.

**Results:**

Four genes—COL1A2, CD163, FN1, and CCL2—were consistently upregulated in both human and murine DN samples. These genes are closely associated with immune activation, ECM remodeling, and chronic inflammation. GSEA revealed their significant enrichment in pathways such as NOD-like receptor signaling, ECM–receptor interaction, and T/B cell receptor signaling, highlighting their potential roles in DN pathogenesis. Experimental validation confirmed elevated expression of these genes at both mRNA and protein levels.

**Conclusion:**

Our study identifies COL1A2, CD163, FN1, and CCL2 as key molecular signatures involved in the immunoinflammatory and fibrotic progression of diabetic nephropathy. These genes hold promise as potential biomarkers and therapeutic targets, offering novel insights into the molecular mechanisms and clinical management of DN.

## 1 Introduction

Diabetic nephropathy (DN) is one of the most common and serious microvascular complications of diabetes mellitus and remains the leading cause of end-stage renal disease (ESRD) worldwide (1, 2). Histopathologically, DN is characterized by glomerular basement membrane thickening, mesangial matrix expansion, tubulointerstitial fibrosis, and persistent proteinuria (3–5). Clinically, DN progresses from an initial stage of hyperfiltration and microalbuminuria to overt proteinuria, declining glomerular filtration rate, and ultimately ESRD, posing a substantial burden on global healthcare systems. Although advances in glycemic control and inhibition of the renin–angiotensin system have improved patient outcomes to some extent, the incidence and progression of DN remain inadequately controlled, suggesting that current therapeutic strategies fail to address the complex underlying molecular mechanisms.

Emerging evidence indicates that DN is not solely driven by metabolic and hemodynamic abnormalities, but also involves chronic inflammation, immune cell infiltration, and dysregulated extracellular matrix (ECM) remodeling (6–9). Despite these findings, the specific molecular mediators and regulatory networks orchestrating these pathological processes remain poorly defined. Moreover, the absence of reliable early diagnostic biomarkers and effective molecular targets continues to hinder timely intervention and personalized therapy. While several bioinformatics-based studies have previously identified hub genes in DN, our work distinguishes itself by integrating two independent human renal transcriptomic datasets with rigorous batch-effect correction, applying a multi-step prioritization pipeline that combines weighted gene co-expression network analysis (WGCNA), high-stringency protein–protein interaction (PPI) network screening, and dual machine-learning algorithms, and validating the results in both type 1 (streptozotocin-induced) and type 2 (db/db) diabetic mouse models. In addition, our analysis specifically focuses on molecular determinants of DN progression rather than onset, thereby addressing a critical but underexplored stage of disease development.

In this study, we aimed to systematically identify key regulatory genes and signaling pathways contributing to the pathogenesis of DN through integrative bioinformatics approaches and experimental validation. By combining transcriptomic data analysis with *in vivo* tissue-level verification in both human and murine models, we sought to uncover robust molecular signatures that may serve as novel diagnostic markers and therapeutic targets for diabetic nephropathy.

## 2 Materials and methods

### 2.1 Data collection and preprocessing

To ensure biological relevance and comparability, only human renal tissue transcriptomic datasets with clearly annotated diabetic nephropathy (DN) and healthy control samples, adequate sample size (>10 per group), and availability of raw expression data were included. Rodent datasets were excluded from the discovery phase to avoid interspecies variability, but animal models were used exclusively for subsequent experimental validation. Two publicly available transcriptomic datasets related to diabetic nephropathy (DN) were obtained from the Gene Expression Omnibus (GEO) database: GSE96804 and GSE30122 (https://www.ncbi.nlm.nih.gov/geo/) (10, 11). Both datasets contained renal tissue samples from patients with DN and healthy controls. Raw expression data were first normalized using quantile normalization to ensure comparability of expression distributions, then log_2_-transformed to stabilize variance. To enable robust cross-dataset comparisons and minimize potential platform-specific biases, batch effects were corrected using the “ComBat” function from the sva package in R. Differentially expressed genes (DEGs) were identified using the “limma” package in R, with DEGs defined as genes meeting |log_2_ fold change| ≥ 1 (i.e., ≥2-fold difference) and Benjamini–Hochberg FDR–adjusted p < 0.05; these thresholds were applied within each dataset prior to downstream network analyses (12).

### 2.2 Weighted gene co-expression network analysis

To identify gene modules significantly associated with DN, WGCNA was independently conducted on each dataset using the “WGCNA” package in R (13). Soft-thresholding powers were chosen to ensure scale-free topology. Module–trait relationships were computed to identify modules most strongly correlated with disease status. Hierarchical clustering and dynamic tree cutting were applied to detect gene modules, and modules with high module–trait correlation coefficients were retained for further analysis. For each gene, module membership (MM) and gene significance (GS) were calculated to assess intramodular connectivity and biological relevance to DN.

### 2.3 Protein–protein interaction network construction and hub gene selection

PPI networks were constructed based on the overlapping DEGs using the STRING database (https://string-db.org/) with a minimum confidence score threshold of 0.9 (14). The resulting network was visualized using Cytoscape software. Two topological analysis methods—Degree and Betweenness centrality—were selected because they represent complementary aspects of network topology: Degree reflects local connectivity, whereas Betweenness captures a node’s role as a bridge in global information flow. Other centrality measures (e.g., Closeness, BottleNeck, MNC, Radiality, Stress) were not included because in scale-free biological networks they often correlate strongly with Degree or Betweenness, potentially introducing redundancy without improving hub gene discrimination. Both measures were applied independently without explicit weighting, and the top 20 genes from each algorithm were considered equally important. The intersection of these two gene sets was used to define a core set of hub gene candidates, thereby capturing both highly connected nodes (Degree) and critical network bridges (Betweenness) while reducing metric-specific bias. Disconnected nodes were excluded, and interaction confidence was restricted to experimentally validated or curated database interactions where available.

### 2.4 Machine learning-based key gene selection

To further prioritize hub genes, two machine learning models—Random Forest (RF) and Support Vector Machine (SVM)—were implemented using the “randomForest” and “e1071” packages in R, respectively (15, 16). Genes that consistently ranked among the top five in both models were selected as key genes for downstream validation. In RF, the MeanDecreaseGini index was used for feature importance ranking, while in SVM, recursive feature elimination with cross-validation was used to ensure model stability and avoid overfitting.

### 2.5 Expression validation and diagnostic evaluation

The expression patterns of the selected key genes were validated in both GSE96804 and GSE30122 datasets. Receiver operating characteristic (ROC) curve analysis was performed using the “pROC” package in R to evaluate the diagnostic potential of each gene, with the area under the curve (AUC) calculated to assess performance (17). ROC analysis was performed independently for each dataset to assess cross-cohort reproducibility. 95% confidence intervals (CIs) were computed using bootstrapping with 1000 iterations.

### 2.6 Functional enrichment analysis

To explore the functional roles of the identified genes, Gene Set Enrichment Analysis (GSEA) was conducted using the GSE96804 dataset (18). KEGG pathway gene sets (c2.cp.kegg.v7.5.1) were downloaded from the Molecular Signatures Database (MSigDB) and used as the reference. Pathways with a nominal p-value < 0.05 and false discovery rate (FDR) < 0.25 were considered significantly enriched. All genes were pre-ranked based on fold change, and enrichment scores were computed using 1000 permutations.

### 2.7 Human tissue collection and validation

Human renal biopsy samples from patients with pathologically confirmed DN and age- and sex-matched healthy controls were obtained from a local clinical biobank, with appropriate ethical approvals. A total of 10 specimens were analyzed, including 5 DN patients and 5 healthy controls. Inclusion criteria for DN samples were: (i) histologically confirmed diabetic nephropathy, (ii) clinical diagnosis of type 2 diabetes mellitus, and (iii) availability of sufficient renal tissue for both IHC and RNA extraction. Exclusion criteria included coexisting renal pathologies (e.g., IgA nephropathy), acute kidney injury, or systemic autoimmune/inflammatory conditions. Histological confirmation of DN was performed on renal biopsy sections using hematoxylin and eosin (H&E), periodic acid–Schiff (PAS), and Masson’s trichrome staining, and evaluated for characteristic features including mesangial expansion, glomerulosclerosis, and tubulointerstitial fibrosis. All slides were independently reviewed by two board-certified renal pathologists to ensure diagnostic accuracy. Immunohistochemistry (IHC) and quantitative real-time PCR (qPCR) were conducted to assess protein and mRNA expression levels of the key genes. Total RNA was extracted using TRIzol reagent, reverse-transcribed with oligo(dT) primers, and quantified using SYBR Green chemistry. IHC staining intensity was scored semi-quantitatively by two independent pathologists in a blinded manner. All procedures were performed in compliance with institutional ethical guidelines.

Species-specific primers were designed for human and mouse orthologs of COL1A2, CD163, FN1, and CCL2. Primer specificity was confirmed by melt-curve analysis and gel electrophoresis. The sequences used are as follows:

Primers for Human Samples:

COL1A2
Forward: 5′-AGGGCCAAGACGAAGACATC-3′.
Reverse: 5′-CTTGCCCCATTCATTTGTCT-3′.
CD163
Forward: 5′-CCAGTCTCAGTGGTCCTGTC-3′.
Reverse: 5′-GGTAGTCTGCTGGTGATGGA-3′.
FN1
Forward: 5′-GGCTCAGTGGGAACATCAAG-3′.
Reverse: 5′-CTGAGGTTGTTGGTGATGCT-3′.
CCL2
Forward: 5′-CCCAATGAGTAGGCTGGAGA-3′.
Reverse: 5′-TCTGGACCCATTCCTTCTTG-3′.
Primers for Mouse Samples:
Col1a2
Forward: 5′-TGGAGAGAGCATGACCGATG-3′.
Reverse: 5′-CTGTTGCAGTGGTAGGTGATG-3′.
Cd163
Forward: 5′-AGCTGGGATGACTTCCCTAC-3′.
Reverse: 5′-GAGACAGGTCCTTGGTTGGT-3′.
Fn1
Forward: 5′-ATGTGGACCCCTCCTGATAGT-3′.
Reverse: 5′-TTGTAGGTGAATCGCAGGTCA-3′.
Ccl2 (Mcp-1)
Forward: 5′-AGGTCCCTGTCATGCTTCTG-3′.
Reverse: 5′-TCTGGACCCATTCCTTCTTG-3′.

### 2.8 Animal models and experimental validation

Two diabetic mouse models were utilized for *in vivo* validation: streptozotocin (STZ)-induced diabetic mice and db/db genetically diabetic mice. The STZ model was chosen to represent early-stage DN pathology, while the db/db model was selected to reflect advanced-stage DN, enabling assessment of gene expression changes across disease progression. The STZ model recapitulates key features of type 1 diabetes mellitus through selective pancreatic β-cell destruction, whereas db/db mice serve as a spontaneous model of type 2 diabetes characterized by obesity, insulin resistance, and progressive nephropathy. The inclusion of both models enables assessment of gene expression across distinct diabetic pathophysiologies, thereby enhancing the translational relevance of the findings. Following confirmation of DN phenotypes, renal tissues were harvested. IHC and qPCR were used to quantify gene expression at both the protein and transcript levels. STZ was administered via intraperitoneal injection at 50 mg/kg for five consecutive days. db/db mice were monitored until 12 weeks of age. Blood glucose levels and urinary albumin excretion were measured to confirm DN phenotype. All animal experiments were approved by the Institutional Animal Care and Use Committee and conducted according to established animal welfare guidelines.

## 3 Results

### 3.1 Identification of DN–associated gene modules and candidate genes

**Figure 1:**
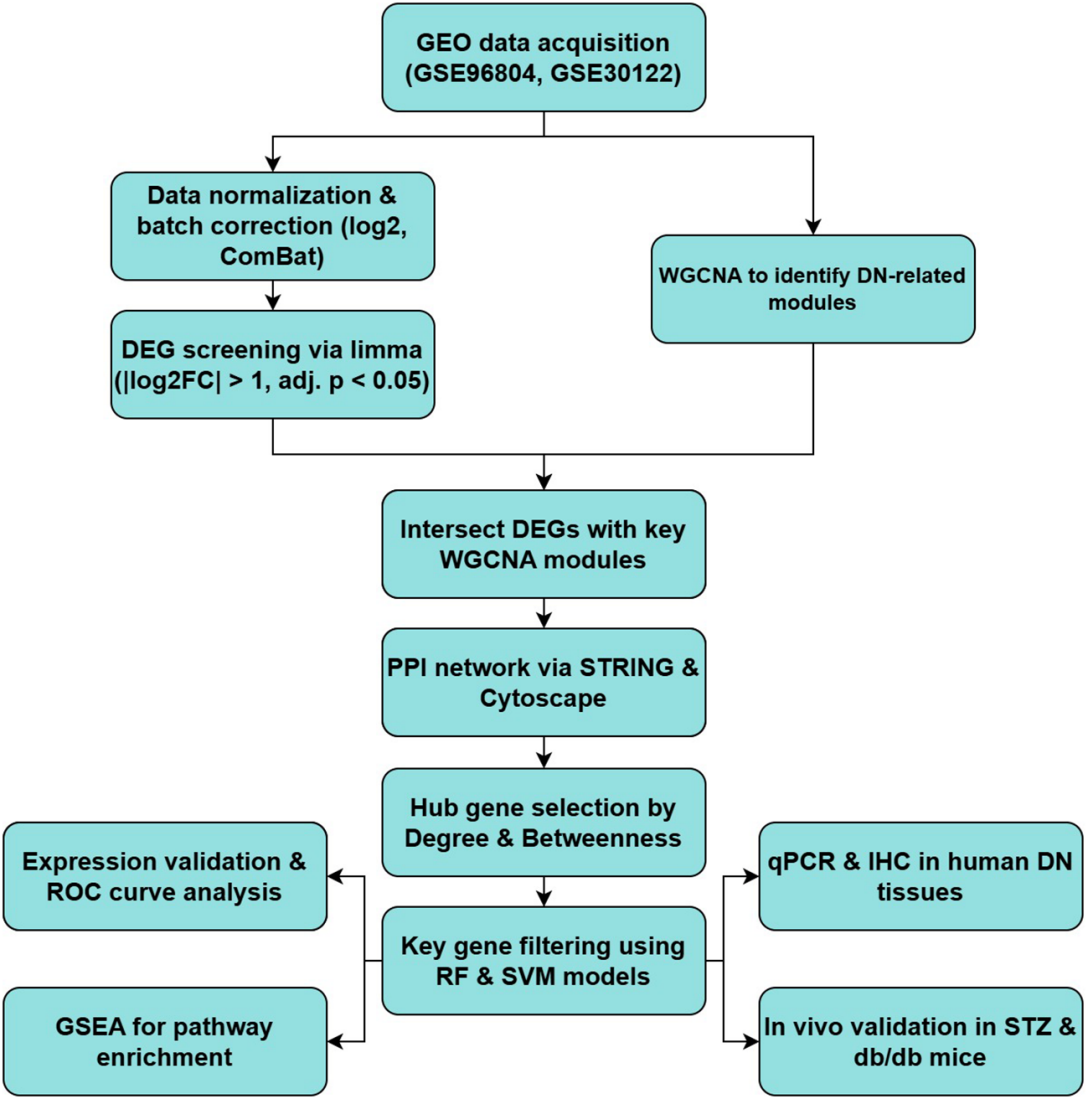
Summary of the bioinformatics and experimental workflow for identifying and validating key genes in DN.

The overall workflow of our integrative analysis is summarized in **Figure 1**, outlining the major analytical and experimental steps performed in this study.

**Figure 2:**
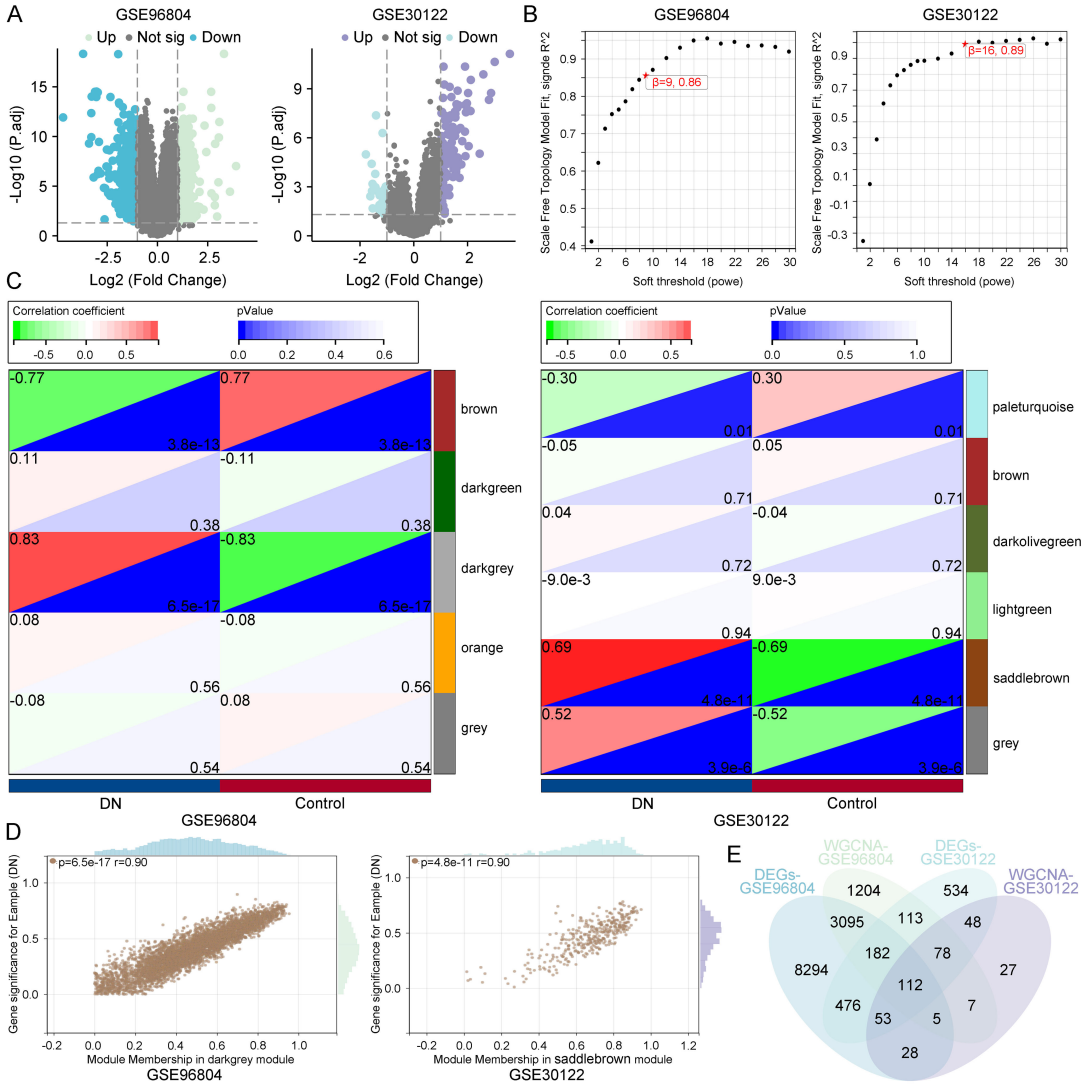
Identification of diabetic nephropathy–associated gene modules. **(A)** Volcano plots of DEGs in GSE96804 and GSE30122 datasets. **(B)** Scale-free topology analysis for selecting soft-thresholding powers in WGCNA. **(C)** Module–trait relationships indicating the darkgrey module in GSE96804 and the saddlebrown module in GSE30122 as most significantly correlated with diabetic nephropathy. **(D)** Correlation between MM and GS within key modules. **(E)** Venn diagram identifying 112 overlapping genes from DEGs and key co-expression modules across datasets.

To identify genes associated with diabetic nephropathy (DN), differential expression analysis was conducted on the GSE96804 and GSE30122 datasets. A substantial number of differentially expressed genes (DEGs) were identified in both datasets using the thresholds of |log2 fold change| > 1 and adjusted p-value < 0.05, as illustrated by the volcano plots (**Figure 2A**).

Subsequently, weighted gene co-expression network analysis (WGCNA) was applied to each dataset to identify gene modules correlated with DN status. Based on scale-free topology criteria, soft-thresholding powers of 9 (GSE96804) and 16 (GSE30122) were selected to ensure robust network construction (**Figure 2B**). Module–trait relationship analysis revealed that the darkgrey module in GSE96804 (correlation = 0.83, p = 6.5e-17) and the saddlebrown module in GSE30122 (correlation = 0.69, p = 4.8e-11) were most strongly associated with DN (**Figure 2C**), suggesting their biological relevance in disease pathogenesis. Further analysis showed a strong positive correlation between module membership (MM) and gene significance (GS) within these modules (r = 0.90), indicating that genes with high intramodular connectivity are also those most relevant to DN (**Figure 2D**).

To refine the pool of candidate genes, we intersected the DEGs with genes from the disease-associated modules, identifying 112 overlapping genes (**Figure 2E**). These genes represent high-confidence candidates that may play key roles in DN pathophysiology.

### 3.2 Identification of hub and key genes through PPI network and machine learning algorithms

**Figure 3:**
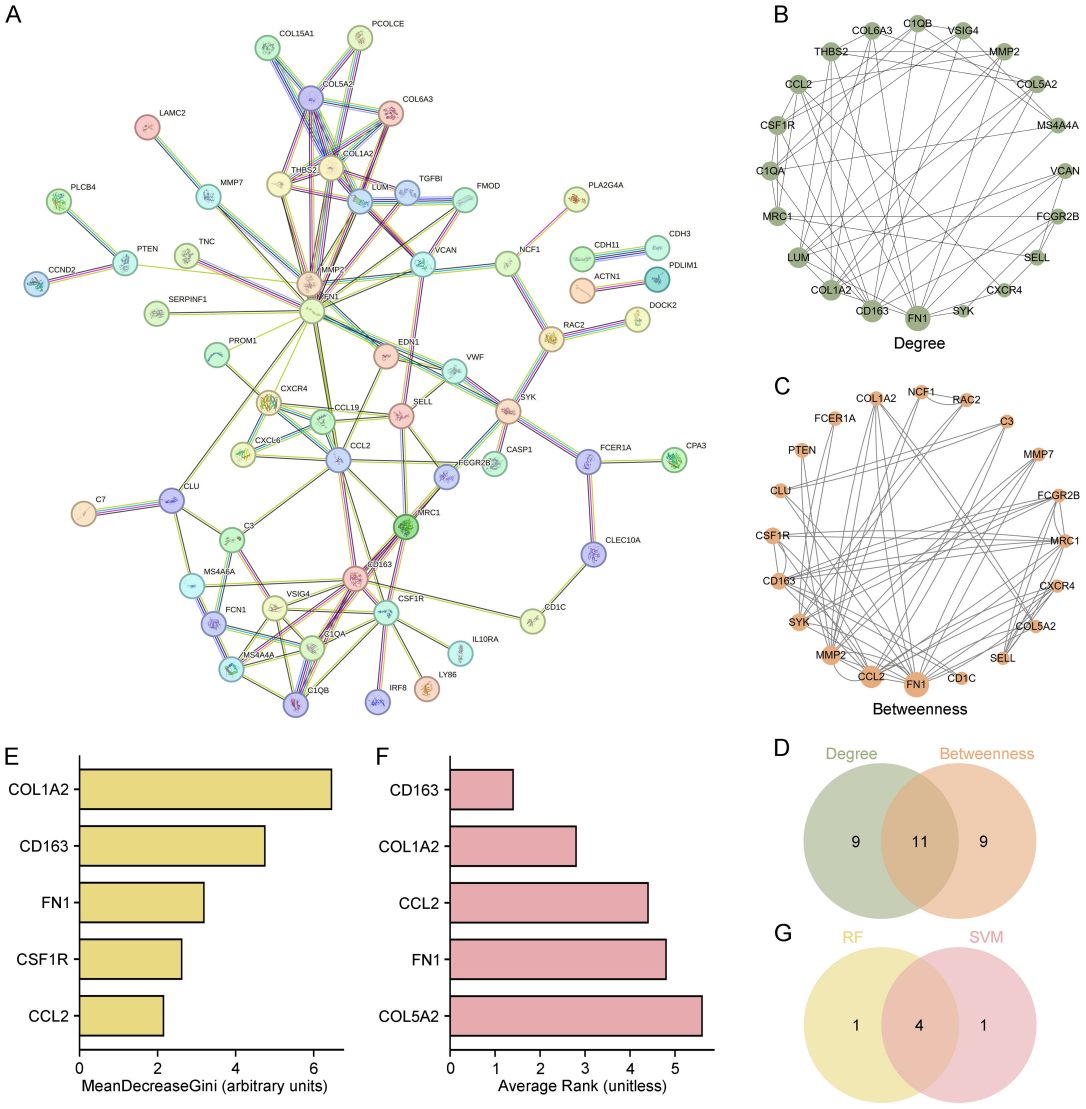
Identification of hub and key genes through PPI network and machine learning. **(A)** PPI network of 112 overlapping genes constructed via STRING. **(B, C)** Top 20 hub genes ranked by Degree and Betweenness centrality metrics using Cytoscape. **(D)** Venn diagram identifying 11 overlapping hub genes shared by both centrality measures. **(E, F)** Top 5 key genes identified by RF and SVM algorithms. **(G)** Venn diagram identifying 4 common key genes (COL1A2, CD163, FN1, and CCL2) shared by RF and SVM.

To further elucidate the molecular mechanisms underlying DN, protein–protein interaction (PPI) analysis was conducted on the 112 overlapping genes using the STRING database. The resulting interaction network showed extensive connectivity, indicating functional interdependence among these genes (**Figure 3A**). Topological analysis using Cytoscape software applied two centrality metrics—Degree and Betweenness—to rank gene importance. The top 20 genes identified by each metric are shown in **Figures 3B** and 3C. A Venn diagram revealed 11 overlapping hub genes, suggesting these as potential network regulators in DN-related networks (**Figure 3D**).

To prioritize the most biologically and clinically relevant targets, two machine learning models—Random Forest (RF) and Support Vector Machine (SVM)—were used. RF analysis identified COL1A2, CD163, FN1, CSF1R, and CCL2 as the top five genes based on MeanDecreaseGini (**Figure 3E**), while SVM ranked CD163, COL1A2, CCL2, FN1, and COL5A2 as top candidates (**Figure 3F**). Intersection of these two models revealed four common genes—COL1A2, CD163, FN1, and CCL2—which were designated as key molecular signatures (**Figure 3G**).

These four genes are proposed to play central roles in DN pathogenesis, particularly in immune regulation and extracellular matrix (ECM) remodeling, and may serve as potential biomarkers or therapeutic targets.

### 3.3 Expression validation and diagnostic evaluation of key genes

**Figure 4:**
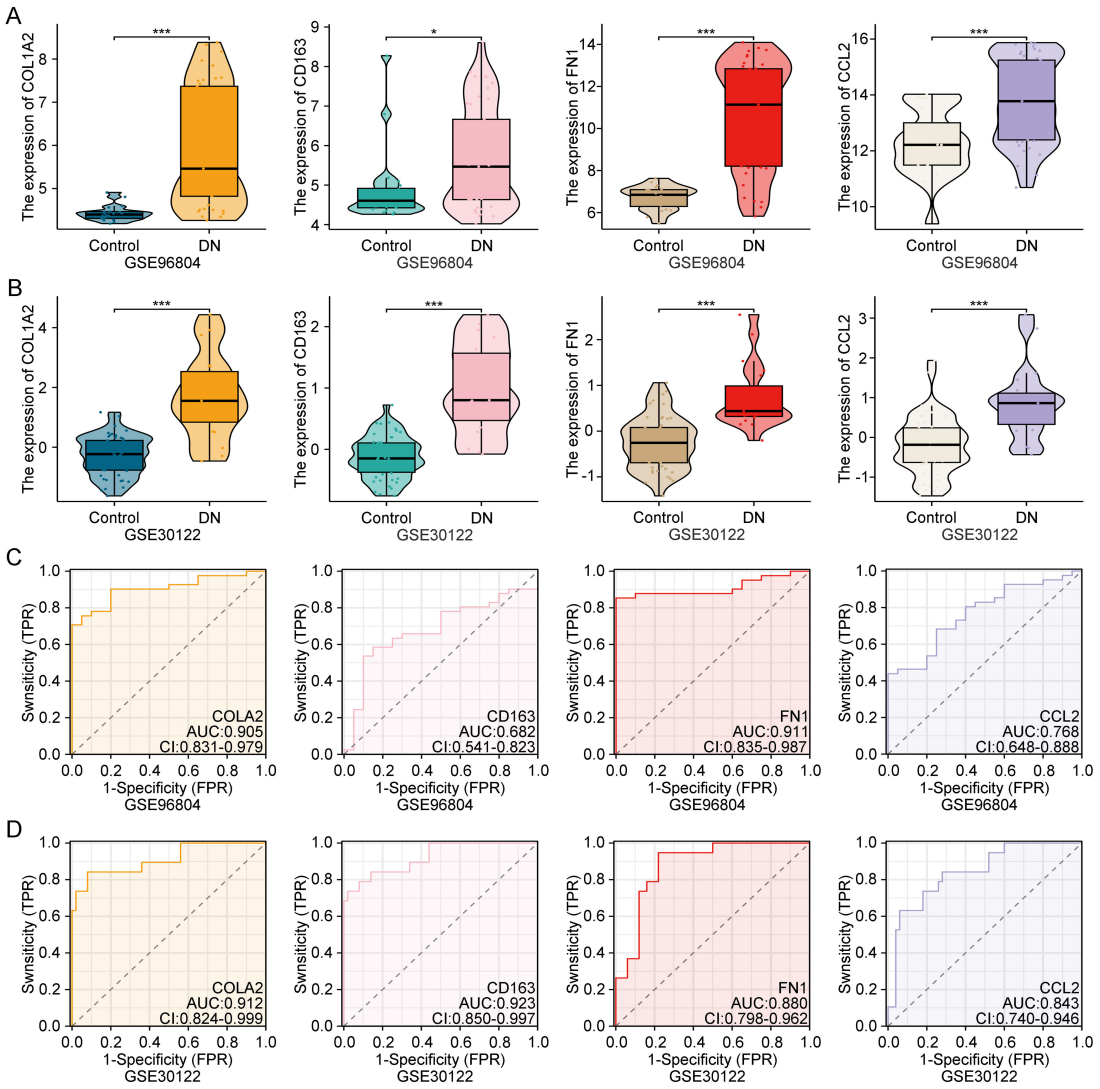
Expression validation and diagnostic performance of key genes in public datasets. **(A, B)** Expression levels of COL1A2, CD163, FN1, and CCL2 in GSE96804 and GSE30122, showing significant upregulation in diabetic nephropathy samples. **(C, D)** ROC curves showing the diagnostic accuracy of each gene in both datasets. **Statistical significance is indicated as follows: *p < 0.05, ***p < 0.001.

To validate the biological significance of COL1A2, CD163, FN1, and CCL2, their expression levels were analyzed in both the GSE96804 and GSE30122 datasets. As shown in **Figures 4A, B**, all four genes were significantly upregulated in DN samples compared to healthy controls (p < 0.05), indicating consistent dysregulation in diseased renal tissues.

Receiver operating characteristic (ROC) curve analysis was further performed to assess their diagnostic performance. All four genes demonstrated moderate to high area under the curve (AUC) values in both datasets (**Figures 4C, D**), suggesting reliable discriminatory power for DN. These findings confirm that these genes may serve as robust transcriptomic biomarkers for early DN detection and clinical stratification.

### 3.4 Functional enrichment analysis of key genes

**Figure 5:**
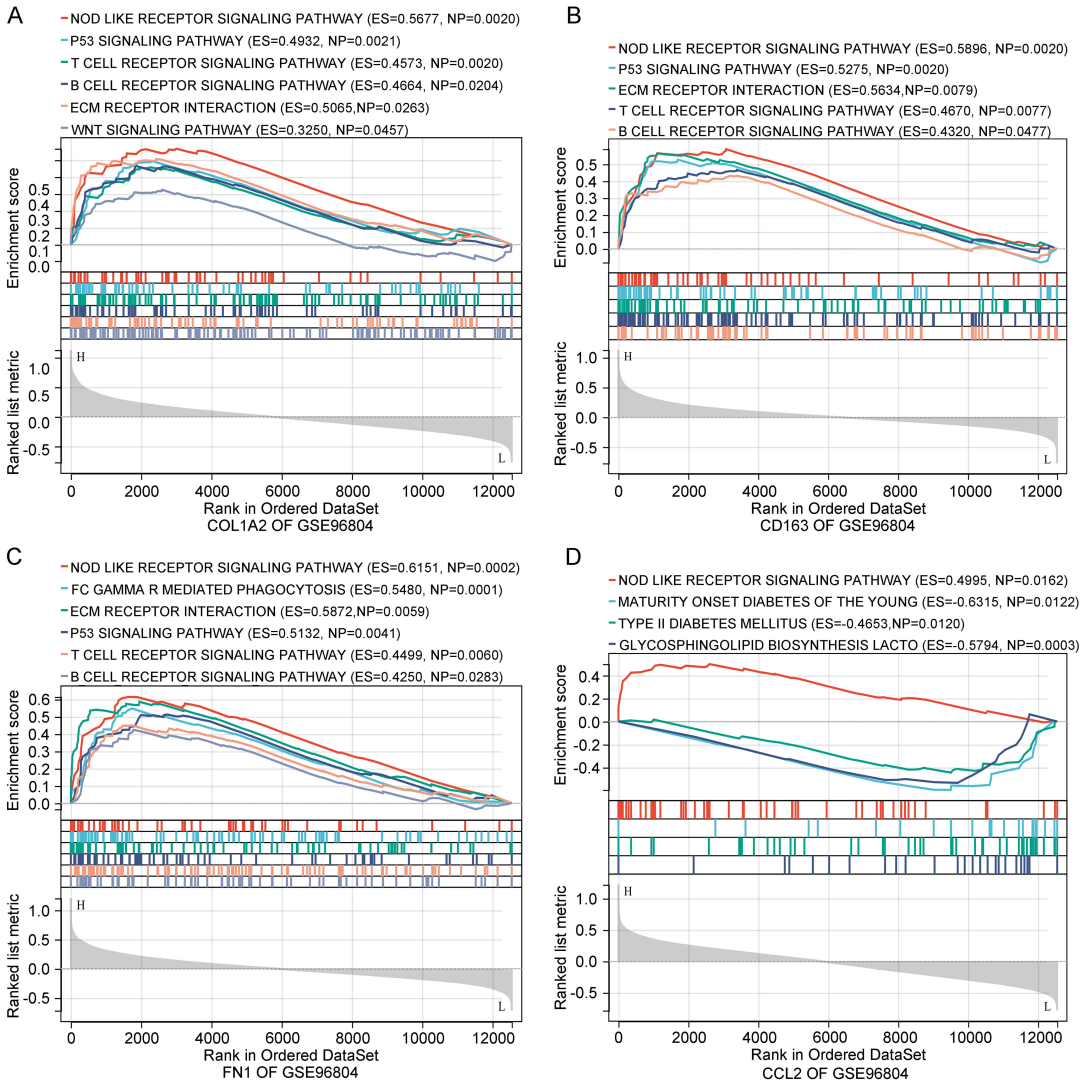
Functional enrichment analysis of key genes. **(A–D)** GSEA of COL1A2, CD163, FN1, and CCL2 based on GSE96804. Enriched pathways involve immune responses, ECM remodeling, and metabolic processes relevant to diabetic nephropathy.

To explore the functional roles of the identified genes, gene set enrichment analysis (GSEA) was conducted using the GSE96804 dataset. The results revealed that COL1A2, CD163, FN1, and CCL2 were significantly enriched in several immune- and inflammation-related pathways (**Figures 5A–D**). Among the most consistently enriched pathways was the NOD-like receptor signaling pathway, implicating innate immune activation in DN. Additionally, pathways such as ECM–receptor interaction, T cell receptor signaling, B cell receptor signaling, and the p53 signaling pathway were enriched for COL1A2, FN1, and CD163, suggesting involvement in ECM remodeling, immune cell activation, and cellular stress responses.

Notably, CCL2 was uniquely enriched in glycosphingolipid biosynthesis and type I diabetes mellitus pathways, indicating its potential role in bridging metabolic and inflammatory processes. Collectively, these enrichment results support the idea that the key genes contribute to DN progression through coordinated regulation of immune, inflammatory, and ECM-related mechanisms.

### 3.5 Validation of key gene expression in human diabetic nephropathy tissues

**Figure 6:**
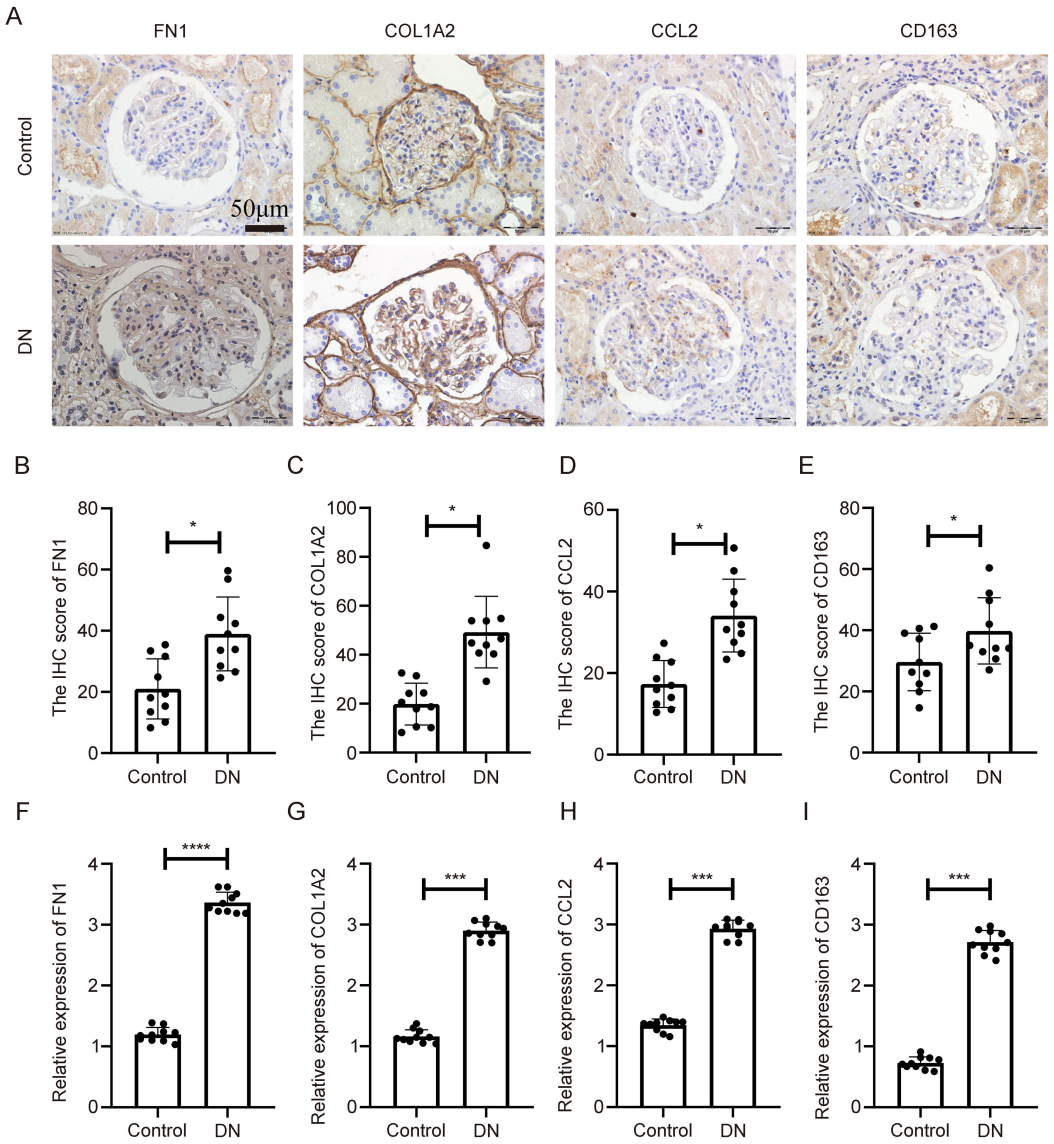
Expression validation of key genes in human diabetic nephropathy tissues. **(A)** IHC staining of COL1A2, CD163, FN1, and CCL2 in kidney tissues from diabetic nephropathy patients and controls. **(B–E)** Quantitative analysis of IHC staining intensity. **(F–I)** mRNA expression levels of the four genes assessed by qPCR in the same human samples. **Statistical significance is indicated as follows: *p < 0.05, ***p < 0.001, ****p < 0.0001.

To confirm the clinical relevance of the identified genes, immunohistochemical (IHC) staining was performed on renal tissues from DN patients and matched healthy controls. As shown in **Figure 6A**, all four genes exhibited markedly elevated protein expression in DN samples, primarily localized in glomerular and tubular compartments. Semiquantitative analysis revealed statistically significant differences in staining intensity between DN and control tissues (**Figures 6B–E**, p < 0.05).

Quantitative real-time PCR (qPCR) was also conducted on the same tissue samples. As shown in **Figures 6F–I**, mRNA levels of COL1A2, CD163, FN1, and CCL2 were significantly upregulated in DN tissues compared to controls (p < 0.05). These findings confirm disease-specific overexpression of the key genes at both transcript and protein levels, reinforcing their relevance as clinical biomarkers.

### 3.6 *In vivo* validation in diabetic nephropathy mouse models

To further validate gene expression *in vivo*, two diabetic mouse models were employed: STZ-induced diabetic mice and genetically diabetic db/db mice.

**Figure 7:**
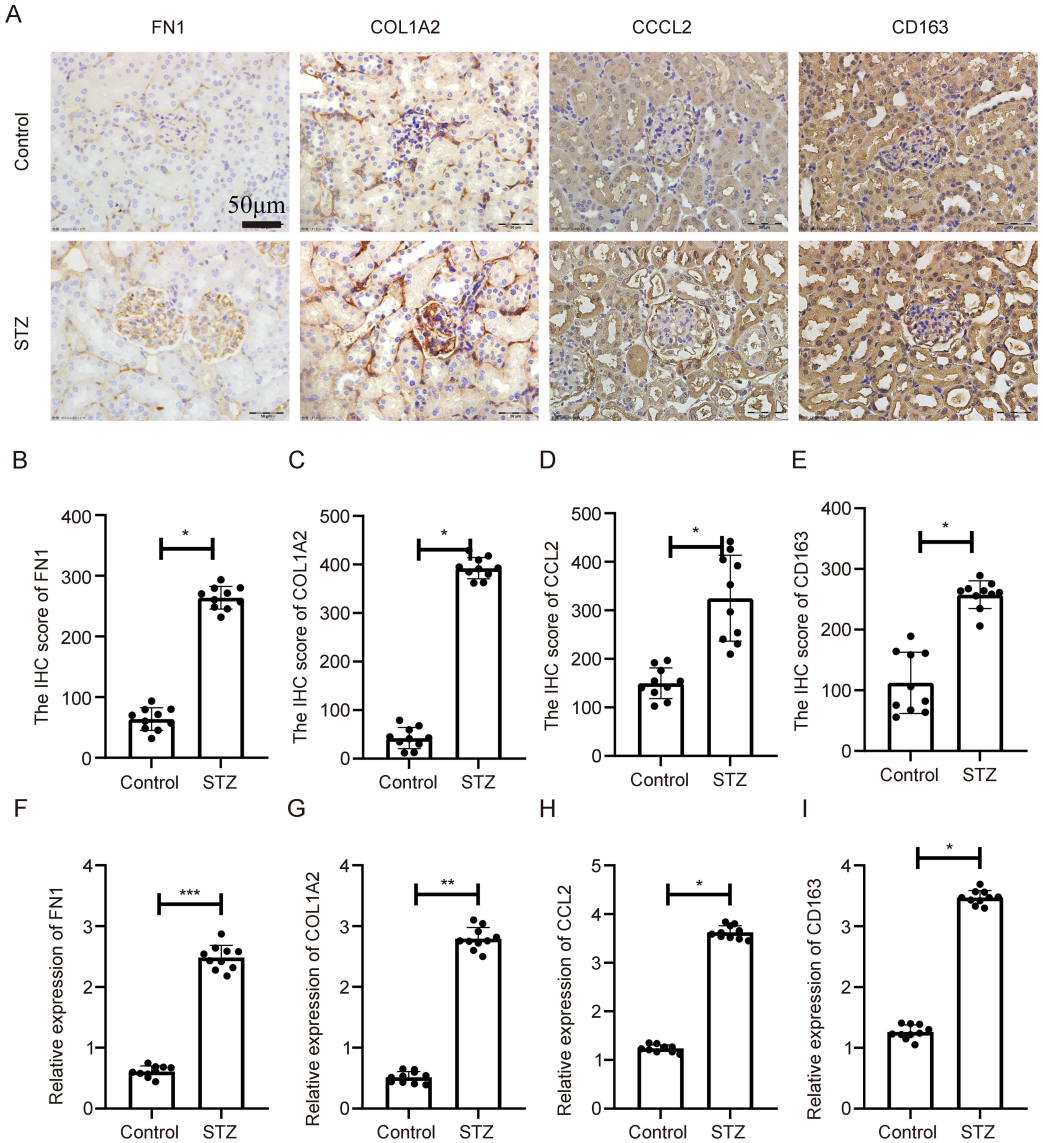
Expression validation of key genes in STZ-induced diabetic nephropathy mouse model. **(A)** IHC showing elevated expression of COL1A2, CD163, FN1, and CCL2 in renal tissues of STZ-treated mice. **(B–E)** Statistical quantification of IHC results. **(F–I)** qPCR results showing significant transcriptional upregulation of the four genes. **Statistical significance is indicated as follows: *p < 0.05, **p < 0.01, ***p < 0.001.

In the STZ-induced model, IHC analysis revealed significant upregulation of COL1A2, CD163, FN1, and CCL2 in diabetic kidneys compared to controls, with expression predominantly in glomerular and tubulointerstitial regions (**Figure 7A**). Quantitative analysis confirmed this upregulation at the protein level (**Figures 7B–E**, p < 0.05). Corresponding qPCR results also showed significantly increased mRNA levels of all four genes (**Figures 7F–I**, p < 0.05).

**Figure 8:**
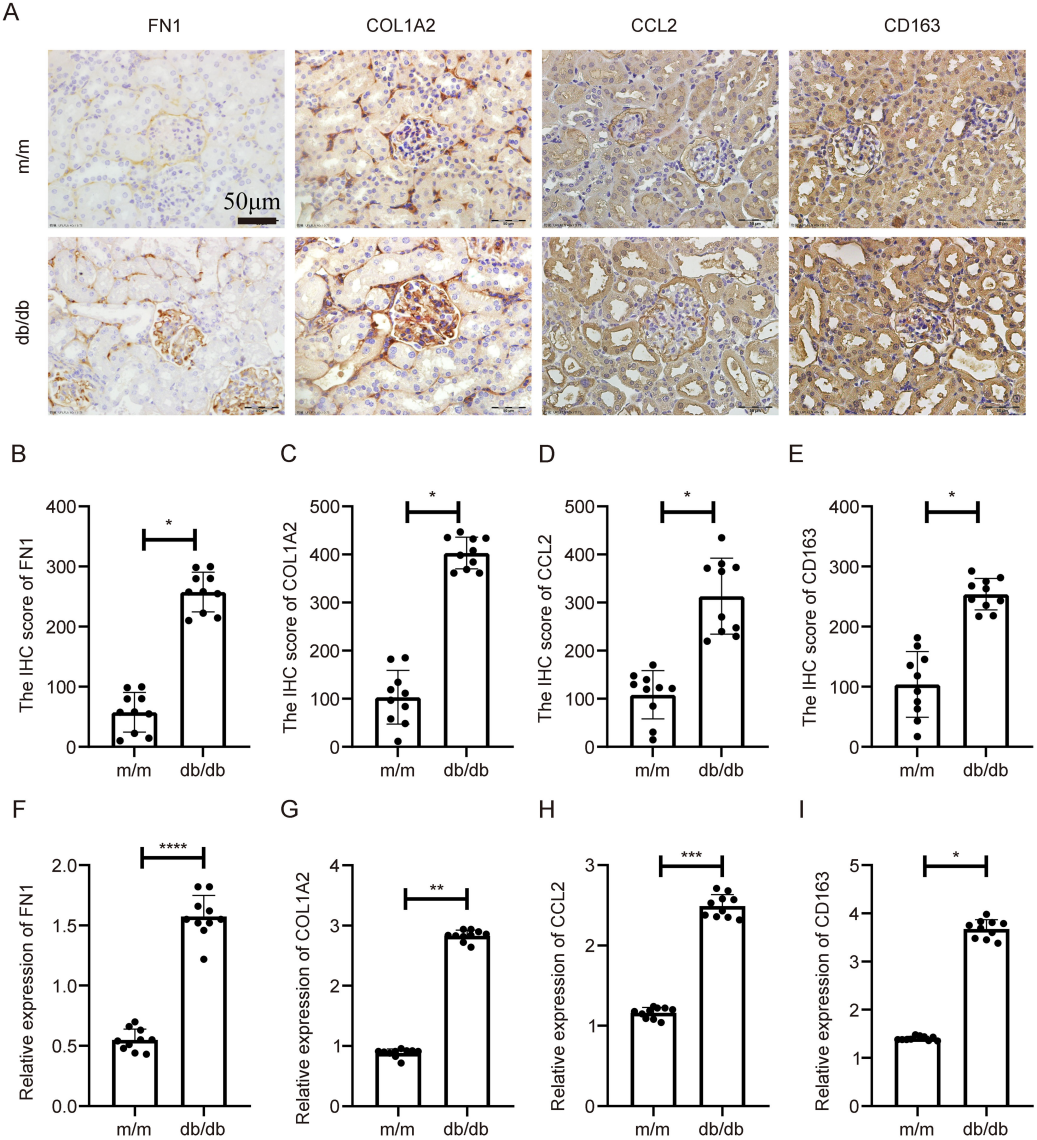
Expression validation of key genes in db/db diabetic nephropathy mouse model. **(A)** IHC staining indicating increased expression of COL1A2, CD163, FN1, and CCL2 in db/db mouse kidneys compared to controls. **(B–E)** Statistical quantification of IHC results. **(F–I)** qPCR results showing significant transcriptional upregulation of the four genes. **Statistical significance is indicated as follows: *p < 0.05, **p < 0.01, ***p < 0.001, ****p < 0.0001.

In the db/db model, a spontaneous model of type 2 diabetes, similar expression patterns were observed. IHC showed increased expression of the four genes in db/db kidneys relative to wild-type controls (**Figure 8A**), with quantification again showing significant differences (**Figures 8B–E**, p < 0.05). qPCR analysis confirmed their transcriptional overexpression in db/db mice (**Figures 8F–I**, p < 0.05).

These consistent findings across both models demonstrate that COL1A2, CD163, FN1, and CCL2 are reproducibly upregulated in DN, supporting their mechanistic roles in diabetic renal injury across different pathological contexts.

## 4 Discussion

DN is a chronic microvascular complication and remains a leading cause of ESRD, characterized by progressive glomerulosclerosis, persistent inflammation, and ECM accumulation (19). In this study, we identified four robust DN-associated genes—COL1A2, CD163, FN1, and CCL2—through a comprehensive strategy integrating transcriptomic analysis, network-based screening, machine learning algorithms, and experimental validation. These genes were consistently upregulated in both human kidney tissues and diabetic mouse models and demonstrated strong discriminatory power in independent public datasets, supporting their potential utility as reliable molecular biomarkers and therapeutic targets for DN. Notably, their functional annotation and pathway enrichment profiles suggest that these genes collectively may drive key pathogenic processes in DN, including extracellular matrix remodeling and fibrosis (COL1A2, FN1), immune cell recruitment and chronic inflammation (CCL2, CD163), oxidative stress–induced injury, and metabolic reprogramming of renal parenchymal cells under hyperglycemic stress.

COL1A2 encodes the α2 chain of type I collagen, a major structural component of the ECM (20). Excessive type I collagen deposition is a hallmark of renal fibrosis, a central pathological feature of DN (21). Prior studies have shown that COL1A2 expression is upregulated in fibrotic kidneys and is regulated by TGF-β1 signaling (22). Its overexpression in our study suggests a pivotal role in mesangial matrix expansion and tubulointerstitial fibrosis, potentially reflecting maladaptive tissue remodeling responses in diabetic kidneys.

CD163 is a scavenger receptor primarily expressed on M2-polarized macrophages, which are typically associated with anti-inflammatory and tissue-repair functions (23). Elevated CD163 levels have been observed in several chronic kidney diseases, including glomerulonephritis and proteinuric nephropathies (24). In the context of DN, increased infiltration of CD163^+^ macrophages may represent a compensatory anti-inflammatory mechanism or may paradoxically contribute to low-grade chronic inflammation and progressive fibrosis. Its consistent upregulation across models and human samples may serve as a marker of immune dysregulation in diabetic kidneys. Notably, CD163 is a classical marker of alternatively activated (M2) macrophages, which have been shown to mediate anti-inflammatory responses and tissue repair in diabetic nephropathy. However, persistent activation of M2 macrophages may paradoxically contribute to chronic inflammation and fibrosis in late-stage disease. This duality is consistent with previously described anti-inflammatory mechanisms in DN that ultimately fail to resolve inflammation, shifting toward a profibrotic immune microenvironment. Additionally, enrichment of the p53 pathway—particularly associated with COL1A2 and CD163—may reflect a maladaptive stress response involving apoptosis and immune modulation, further linking our findings to known immune-regulatory axes in DN.

FN1, encoding fibronectin, is a key ECM glycoprotein involved in cell adhesion, tissue remodeling, and fibrogenesis (25). Accumulation of fibronectin in glomerular and interstitial regions is well documented in DN, where it contributes to glomerular basement membrane thickening and vascular occlusion (26). Our findings further support FN1 as a core contributor to ECM remodeling in DN pathology.

CCL2 (also known as MCP - 1) is a chemokine that plays a critical role in monocyte and macrophage recruitment to sites of inflammation (27). Its elevated expression in DN has been linked to proteinuria and glomerular damage (28). Given its chemotactic role in immune cell trafficking, CCL2 upregulation may contribute directly to immune infiltration and sustained renal inflammation in DN.

To further address the immunological relevance of these genes, we note that COL1A2 and FN1 can modulate immune activation through ECM–integrin signaling, influencing leukocyte adhesion and migration. CD163 is functionally involved in the clearance of inflammatory hemoglobin–haptoglobin complexes and reflects macrophage-mediated immune remodeling. CCL2 is a well-characterized chemokine essential for monocyte recruitment and has been extensively implicated in diabetic renal immune injury.

To gain insight into the broader biological roles of these genes, we performed GSEA. All four genes were significantly enriched in the NOD-like receptor signaling pathway, which is implicated in renal inflammation through activation of the NLRP3 inflammasome, resulting in IL - 1β production and podocyte injury (29). This supports the critical contribution of innate immunity to DN progression.

Interestingly, enrichment of the T cell receptor and B cell receptor signaling pathways indicates a potential role of adaptive immunity, which has often been underappreciated in DN pathogenesis. Accumulating evidence suggests that T and B lymphocytes infiltrate diabetic kidneys and promote injury via cytokine production and antigen presentation (30, 31). The enrichment of these pathways further supports the immunological significance of the identified genes in modulating both innate and adaptive immune responses in DN.

Additionally, the ECM–receptor interaction pathway was significantly enriched, highlighting interactions between ECM components and integrins that regulate fibrosis and cellular adhesion (32). This finding aligns with the fibrotic phenotype of DN and supports the involvement of FN1 and COL1A2 in matrix-driven kidney damage. Enrichment of the p53 signaling pathway for COL1A2 and CD163 further suggests roles in apoptosis and cellular senescence under hyperglycemic stress (33), processes known to contribute to tubular injury and DN progression.

Importantly, among the four identified DN-associated genes, CCL2 and FN1 represent pharmacologically tractable targets with direct translational potential. CCL2 is a key chemokine in monocyte recruitment, and inhibitors of the CCL2–CCR2 axis (e.g., bindarit, CCX872) have demonstrated renoprotective effects in diabetic kidney disease by reducing macrophage infiltration and inflammation. FN1 can be indirectly targeted through inhibition of fibronectin–integrin signaling, such as with α5β1 integrin antagonists, which have shown anti-fibrotic activity in experimental nephropathy. Although COL1A2 and CD163 currently lack direct approved inhibitors, COL1A2 can be modulated via upstream TGF-β/SMAD blockade—a validated anti-fibrotic strategy—while CD163 may serve as a candidate for macrophage-targeted drug delivery systems. These insights not only underscore the therapeutic relevance of our findings but also strengthen their translational value in DN management.

Despite the strength of our integrative approach and validation across species, several limitations must be acknowledged. First, the transcriptomic data utilized were derived from bulk tissue, which precludes resolution of cell-type-specific expression patterns. The use of single-cell RNA sequencing or spatial transcriptomics in future studies would help localize gene expression more precisely. Second, although expression levels were validated across datasets and through mRNA/protein analyses, direct functional validation was not performed. Specifically, functional perturbation experiments—such as gene knockdown, overexpression, or pharmacologic inhibition—were not conducted, limiting our ability to confirm the causal involvement of these genes in DN pathogenesis. Third, the absence of clinical correlation analyses—such as associations with estimated glomerular filtration rate (eGFR), proteinuria, or disease stage—limits the direct translational applicability of these markers. Finally, as with most transcriptomics-based integrative analyses, our approach cannot definitively distinguish true disease-driving genes from reactive or bystander transcripts. While our use of multiple independent selection layers increases robustness, functional studies such as gene perturbation assays are essential to validate causality and therapeutic relevance.

In conclusion, our study identifies COL1A2, CD163, FN1, and CCL2 as key molecular players involved in fibrosis, immune activation, and inflammatory signaling in diabetic nephropathy. By linking these molecular alterations to well-established pathological hallmarks—fibrotic ECM deposition, sustained immune activation, oxidative stress responses, and dysregulated metabolic pathways—our findings provide a plausible mechanistic framework for their contribution to DN progression. Their consistent dysregulation, enrichment in biologically relevant pathways, and validation in both human and animal models highlight their potential as clinically relevant biomarkers and therapeutic targets. Future investigations focusing on mechanistic validation, cellular localization, and clinical stratification will be essential to translate these findings into meaningful clinical applications.

## 5 Conclusions

In this study, we identified COL1A2, CD163, FN1, and CCL2 as key genes strongly associated with the progression of DN. These genes are implicated in fundamental pathological processes, including extracellular matrix deposition, immune cell infiltration, and chronic inflammation. Their consistent overexpression across multiple human datasets and diabetic mouse models, combined with enrichment in biologically relevant signaling pathways, underscores their potential as robust biomarkers and promising therapeutic targets. These findings provide valuable insights into the molecular mechanisms underlying DN and offer a foundation for the development of early diagnostic tools and targeted interventions in diabetic kidney disease.

## Data Availability

The original contributions presented in the study are included in the article/supplementary material. Further inquiries can be directed to the corresponding authors.
